# Use of the internet by Italian pediatricians: habits, impact on clinical practice and expectations

**DOI:** 10.1186/1472-6947-12-23

**Published:** 2012-03-28

**Authors:** Mariateresa Romano, Francesco Gesualdo, Elisabetta Pandolfi, Alberto E Tozzi, Alberto G Ugazio

**Affiliations:** 1 Bambino Gesù Children's Hospital, Piazza Sant'Onofrio 4, Rome, Italy

## Abstract

**Background:**

Medical professionals go online for literature searches and communication with families.

We administered a questionnaire to members of the Italian Society of Pediatrics to assess determinants of their use of the Internet, of social platforms and of personal health records during clinical practice.

**Methods:**

All the 9180 members of the Italian Society of Pediatrics were invited to fill in a questionnaire concerning use of the Internet and usefulness of Internet-based tools during clinical practice. The questionnaire was administered through the SurveyMonkey^® ^web platform. Logistic regression analysis was used to study factors affecting use and influence of the Internet in clinical practice.

**Results:**

A total of 1335 (14.5%) members returned the questionnaire. Mean age was 49.2 years, 58.6% were female. 32.3% had access to the Internet through a Smartphone. 71.9% of respondents used the Internet during clinical practice, mainly searching for guidelines and drug references. Use of the Internet during clinical practice was more frequent among younger pediatricians (OR 0.964; 95% CI 0.591-0.978), males (OR 1.602; 95% CI 1.209-2.123) and those living in Northern and Central Italy (OR 1.441; 95% CI 1.111-1.869), while it was lower among family pediatricians. 94.6% of respondents were influenced in their clinical practice by information found on the Internet, in particular younger pediatricians (OR 0.96, 95% CI 0.932-0.989), hospital pediatricians (OR 2.929, 95% CI 1.708-5.024), and other pediatric profiles (OR 6.143, 95%CI 1.848-20.423). 15.9% of respondents stated that social networks may be useful in pediatric practice. Slightly more than half (50.5%) of respondents stated that personal health records may be clinically relevant. Registrars and hospital pediatricians were more likely to perceive personal health records as useful tools for clinical practice. Additional resources pediatricians would like to access were free bibliographic databases and tools for interacting with families.

**Conclusions:**

Italian pediatricians frequently use the Internet during their practice. One-third of them access the Internet through a Smartphone. Interaction with families and their empowerment can be improved by the use of Internet tools, including personal health records, toward which respondents show a significant interest. Though, they show a general resistance to the introduction of social networks in clinical practice.

## Background

Information technology is recognized as a useful tool for improving patient safety and quality of care [1]. The Internet has the potential to improve information dissemination and change the way health care is delivered [2-7].

The vast majority of medical professionals use the Internet for Medline searches [8]. However, they also make use of the Internet for e-mail communication with patients and transmission of test results to improve continuity of care [9].

Social networks may represent a new frontier for health care [10]. By means of social networks, physicians can share medical information with the public, with the potential of a positive impact on health choices and behaviours. On the other hand, conflicts of interest and potential violation of patients' privacy are a matter of concern with the use of this technology [11]. In this respect, the American Medical Association recently issued a policy statement on professionalism in the use of social media by physicians [12], addressing the need monitoring their own presence on the Internet, adopting privacy measures, and maintaining appropriate boundaries in the online relationship with patients.

Personal health records also represent potentially useful tools for improving health care through sharing of information directly recorded by patients. A recent study showed that physicians may be interested in this approach in respect to information portability and engagement of patients in the health care process [13].

A survey published by the European Commission in 2008 reported that, on average, 66% of general practitioners use the Internet during consultation throughout Europe [14] with marked differences among Member States ranging from a minimum of 3% (Latvia) to a maximum of 94% (Estonia).

Few data have been published on the use of the Internet by pediatricians and its influence on clinical decisions. Although the web may not represent the major source of information for general pediatricians, its use in clinical practice is widely documented [15]. A study carried out in the United States showed that results of Internet searches affect general pediatricians' decisions on patient's care in 71.8% of cases [16]. One additional study carried out among Ireland pediatricians showed a frequent use of the Internet for searching medical literature through Pubmed and health information material for patients [17].

Italy has a large community of pediatricians with different professional profiles, including hospital and university pediatricians, private pediatricians, and family pediatricians, the latter providing free primary health care to all resident children.

The majority of Italian pediatricians are members of the Italian Society of Pediatrics, which counts 9180 active members, 83.2% of whom provided a valid email address when subscribing to the Society.

We performed a cross sectional study among its members with the objective of describing the use of the Internet during clinical practice, describing the perception of social networks and personal health records, and exploring sociodemographic and professional variables that influence these outcomes.

## Methods

A cross sectional survey through a standardized questionnaire was performed among members of the Italian Society of Pediatrics in the period February-May 2011.

The study was approved by the Institutional Review Board of the Italian Society of Pediatrics.

### The Italian society of pediatrics

The Italian Society of Pediatrics was founded in 1898. In 2011 it counts 9180 active members, including family pediatricians, pediatricians from Universities and hospitals, pediatricians working in private practices, community pediatricians working in health centers, and registrars in pediatrics. The Society is a no-profit organization, and provides scientific information on pediatrics to institutions, physicians, families and associations. Membership to the Society is voluntary, and open to all registrars and specialists in pediatrics.

### Study population and timeframe

In February 2011, the 7638 members of the Society that had provided a valid email address (83.2% of the total members) were sent an email invitation to participate in the survey, providing a link to a web-based questionnaire. Pediatricians who did not provide a valid email address were significantly older than those who had provided it (52 years ± 10, p > 0.001).

No geographical or other exclusion criteria were applied. In March 2011, one month after the first invitation, a solicitation to join the survey was sent.

An invitation to fill the web-based questionnaire was also enclosed in the April 2011 print issue of the Society's news magazine, which is sent on a monthly base to the regular address of all the 9180 Society members. The survey was closed at the end of May 2011.

### Data collection

The questionnaire was prepared using the web-based SurveyMonkey^® ^platform.

The content of the questionnaire was discussed with the Society's Review Board and with a panel of pediatricians, and finalised after a pilot test in a sample of 10 pediatricians.

Questionnaires were anonymously recorded. We reviewed the IP address of respondents for the exclusion of duplicates.

The questionnaire included questions on: age (continuous); gender (dichotomous); Region of residence (categorical, 2 levels: Northern and Central Italy vs Southern Italy); professional profile (categorical, 5 levels: family pediatrician, hospital pediatrician, University pediatrician, registrar in pediatrics, other; the category "Other" included all job profiles not classifiable in the previous categories, such as private pediatricians and ambulatory specialists working in local health units); Internet access at home (dichotomous); Internet access at work (dichotomous); Internet access through Smartphone (dichotomous); frequency of Internet access (categorical, 5 levels); use of the Internet during practice (i.e.:" "do you use the Internet during clinical practice?"; dichotomous); most searched topics (categorical, 6 levels); most used databases (categorical, 8 levels); influence of information found on the Internet on clinical decisions (i.e.: "do information found on the Internet influence your clinical decisions?"; categorical, 3 levels: always, sometimes, never); usefulness of social networks in clinical practice (i.e.: "do you think that social networks may be useful for clinical practice?"; categorical, 3 levels: yes, no, do not know); usefulness of personal health records in clinical practice ("do you think that sharing clinical information with families through personal health records can improve quality of care?"; categorical, 3 levels: yes, no, do not know); other professional use of the Internet (categorical, 5 levels), additional web resources respondents would like to have on hand for professional use. The questionnaire took 5 minutes on average to be completed during the pilot test.

We included in the final population all the questionnaires that were returned, including those that were not completed.

### Data analysis

Results were analyzed through frequency distribution for discrete variables or means for continuous variables.

For comparison of participants vs. non participants age was compared through the Student's t test; gender was compared through the χ^2 ^test.

We performed logistic regression models to explore the independent effect of age, gender, region of residence and medical profile, adjusting for potential confounding, on the following items: Internet use during clinical practice, influence of the Internet on clinical decisions and perception of usefulness of social networks and personal health records for clinical practice. We excluded from this analysis those who stated that they did not use the Internet during clinical practice.

Age was treated as a continuous variable. Professional profile was treated as a categorical variable (5 levels). Region of residence was treated as a dichotomous variable (Northern and Central regions, Southern regions, according to the National Institute of Statistics categorization [18] As for influence of the Internet on clinical decision, the categories "always" and "sometimes" were grouped in a single category. As for perception of the usefulness of social networks and personal health records for clinical practice, the categories "no" and "do not know" were grouped in a single category. Gender and Internet use during clinical practice were bivariate variables and were treated as such in the analysis.

Regarding reference categories: female and southern regions were used as reference categories for gender and residency, respectively, according to results of a survey conducted by the Italian National Institute for Statistics indicating that these categories have a less frequent access to the Internet [18]. Family pediatrician was arbitrarily used as the reference category for medical profile, under the assumption that use of the Internet by this category of pediatricians during clinical practice may be lower compared to other categories, as they usually deal with less complex pathologies.

As a measure of outcome in logistic regression models, we used adjusted ORs (aORs) and 95% confidence intervals (CI).

STATA 11 statistical software was used for the analysis.

## Results

### Personal information

A total of 1335 members returned the questionnaire (14.5% of all members of the Society). The proportion of missing responses over all the questionnaire items was always less than 2.5%.

General characteristics of the studied population are reported in Table 1.

**Table 1:** Study population

Female sex (N, %)		773 (58.6%)
Age (mean, SD)		49.2 (10.9)

Region (N, %)	North	829 (63.5%)

	South	476 (36.5%)

Professional profile (N, %)	Family pediatrician	401 (29.5%)

	Hospital pediatrician	645 (47.5%)

	University pediatrician	68 (5%)

	Registrar in Pediatrics	68 (5%)

	Other	148 (10.9%)

Mean age of respondents was 49.2 years (SD 10.9; range 26-80), and 773 (58.6%) were females. Compared with the members of the Society who did not participate in the study, our sample included slightly younger individuals (mean age of non participants: 50 ± 8 years, p < 0.01), and a lower proportion of females (females among non participants: 62%; p < 0.01).

Regarding region of residence 829 respondents (63.5%) practiced in the North-Center of Italy, while 476 (36.5%) practiced in the South of the country.

Regarding professional profiles 401 (29.5%) respondents were family pediatricians, 645 (47.5%) were hospital pediatricians, 68 (5%) were University pediatricians, 68 (5%) were registrars, 148 (10.9%) belonged to other professional profiles.

### Access to the Internet and computer use

Almost all respondents had access to the Internet at home (98.2%) and in their office (96.9%), and 32.3% of them had access to the Internet through a Smartphone. Regarding frequency of Internet access, 81% went online daily, and 16% 2-3 times a week. A total of 954 subjects (71.9%) used the Internet during their practice.

### Internet searches

The majority of respondents searched for guidelines (82.7%) and drug references (71.6%). 57.1% and 45.2% of respondents searched for family education and diagnostic support material respectively.

The resources most widely used for medical information seeking during consultations were Pubmed (88.1%) and Cochrane (51.9%) web sites; Google Scholar accounted for 12.7%, Embase for 8.9%, and UpToDate for 8%.

Among interviewed pediatricians, 94.6% answered that their clinical decisions were sometimes or always influenced by information found on the Internet.

In order to assess whether sex, age, region of residence or medical profile affected Internet use during clinical practice, we performed a logistic regression analysis. Results are reported in Table 2. Males, younger individuals and pediatricians living in the Northern and Central part of the country were more likely to use the Internet during their practice. Moreover, University pediatricians, registrars and other medical profiles were more likely to use the Internet during clinical practice compared to family pediatricians.

**Table 2:** Independent effect of age, sex, region of residence and medical profile on use of the Internet during clinical practice (logistic regression model)

Factor	aOR	aOR 95% CI
Age	0.96	0.59-0.98

Gender	1.60	1.21-2.12

Region of residence	1.44	1.11-1.87

Hospital pediatrician	1.42	0.95-2.14

University pediatrician	1.93	1.30-2.87

Registrar	2.53	1.27-5.04

Other	2.43	1.02-5.78

Age was treated as a continuous variable. Reference category for gender was female; reference category for region of residence was Southern regions; reference category for job profile was family pediatrician.

A logistic regression analysis was also performed to assess whether sex, age, region of residence or medical profile affected the influence of information found on the Internet on clinical decisions. Results are reported in Table 3.

**Table 3:** Independent effect of age, sex, region of residence and medical profile on influence of information found on the Internet on clinical decisions (logistic regression model)

Factor	aOR	aOR 95% CI
Age	0.96	0.93-0.99

Gender	1.02	0.60-1.73

Region of residence	0.76	0.45-1.29

Hospital pediatrician	2.80	1.63-4.83

University pediatrician	2.24	0.75-6.70

Registrar	3.16	0.39-25.86

Other	6.16	1.84-20.61

Age was treated as a continuous variable. Reference category for gender was female; reference category for region of residence was Southern regions; reference category for job profile was family paediatrician.

Younger respondents, hospital pediatricians, and other pediatric profiles (including private pediatricians and ambulatory specialists working in local health units) were more likely to be influenced in their clinical decisions by information found on the Internet. No effect of gender was shown by this analysis.

### Social networks, telemedicine and other uses of the Internet

A total of 15.9% of respondents stated that social networks may be useful in pediatric practice. The independent effect of age, sex, region of residence and medical profile on this item is reported in Table 4.

**Table 4:** Independent effect of age, sex, region of residence and medical profile on opinion about usefulness of social networks in pediatric practice (logistic regression model)

Factor	aOR	aOR 95% CI
Age	0.99	0.98-1.01

Gender	1.46	1.05-2.05

Region of residence	0.67	0.49-0.92

Hospital pediatrician	1.26	0.87-1.83

University pediatrician	1.18	0.56-2.49

Registrar	1.85	0.89-3.86

Other	1.18	0.68-2.03

Age was treated as a continuous variable. Reference category for gender was female; reference category for region of residence was Southern regions; reference category for job profile was family paediatrician.

Male respondents and pediatricians working in the North-Center of Italy were more likely to think that social networks may be useful in pediatric practice, while age and medical profile had no effect on the outcome.

A total of 50.5% of respondents stated that sharing clinical information with families through personal health records may be clinically relevant. Table 5 reports the effect of age, gender, region of residence and medical profile on this item.

**Table 5:** Independent effect of age, sex, region of residence and medical profile on opinion about usefulness of personal health records in pediatric practice (logistic regression model)

Factor	aOR	aOR 95% CI
Age	1.00	0.99-1.01

Gender	1.04	0.81-1.33

Region of residence	0.87	0.69-1.10

Hospital pediatrician	1.62	1.24-2.11

University pediatrician	1.38	0.80-2.37

Registrar	5.48	2.75-10.93

Other	1.27	0.86-1.89

Age was treated as a continuous variable. Reference category for gender was female; reference category for region of residence was Southern regions; reference category for job profile was family paediatrician.

While no effect of age, gender and region of residence was found on this outcome, registrars and hospital pediatricians were more likely to perceive personal health records as useful tools for clinical practice.

When asked about other Internet professional uses, respondents answered transmission of laboratory results (57.6%), clinical data exchange with other colleagues for consultation (48.5%), email communication with families (44.2%), and continuing medical education (40.0%).

Finally, respondents provided their opinion on resources they would like to have on hand on the Internet for professional use. Most respondents reported they would like to have free access to bibliographic resources (84.2%). A significant number of respondents were interested in being provided resources for interacting with families, specifically information material (64.4% of respondents), secure communication systems with families (28.9%), personal health records (20.7%), and social communities with families (12.3%). Finally, 47.3% stated they wish to have access to a clinical decision support system through the Internet.

## Discussion

The present study shows that a large majority of Italian pediatricians frequently use the Internet during their practice for supporting their clinical decisions, and are willing to use tools and platforms that favour the interaction with families. The rate of Internet use during consultation detected in our survey parallels that indicated in an European study performed among Italian general practitioners in 2008 [14]. Moreover, our results are in line with those provided by other studies on the use of the Internet during clinical practice by other medical profiles and in other settings [19,20].

Bibliographic resources are among those that medical professionals search most frequently online [21]. The finding that pediatricians search more frequently through Medline and Cochrane is consistent with the results of a previous study performed among general practitioners in Switzerland [22]. Although we did not investigate whether pediatricians preferred to consult open access journals, respondents in our sample clearly expressed the willingness to have a wider access to bibliographic resources. The scientific information need may not be completely satisfied by existing resources. A stronger effort should be put by the scientific community in providing open access resources to physicians.

Access to information on the Internet relevant to clinical practice may however be affected by English skills since a large part of scientific information on the web is provided in this language. Although online translation services have become popular tools, translation in local language of scientific documents, including clinical guidelines, may improve accessibility to their content.

More than 70% of interviewed pediatricians search for clinical guidelines or drug references during their practice, while information concerning diagnosis is searched less frequently. This observation may be relevant to the format in which information is presented on the Internet. In fact, most documents on the Internet are available as plain text, sometimes too long or too complex to be easily managed during practice. Evidence-based information should be presented on the Internet through formats that improve accessibility and readability, provided in conjunction with traditional documents, if they are to be effectively used by clinicians.

We did not investigate how pediatricians seek information for diagnosis. It is possible that diagnostic features are searched online through generic search engines [23]. Although this approach may be helpful, a wider access to clinical decision support systems is desirable and a significant proportion of respondents expressed the willingness to access such systems.

Thirty-two percent of respondents stated they access the Internet through a Smartphone. Italy is one of the countries with the highest Smartphone penetration in the world, with nearly 38% of total users in 2011 [24]. The large use of Smartphones represents an opportunity for improving access to clinically relevant information on the web. At present, medical Smartphone applications are mostly available in English. Availability of this kind of applications in local language is likely to improve access to scientific information useful for clinical practice.

A significant proportion of respondents in our study as well as in other studies [21] is interested in collecting from the Internet medical information material for families. Moreover, the results of our study show that information material for families, secure communication systems with families, personal health records, and social platforms are desirable web resources for pediatricians. This observation confirms the interest of pediatricians for tools that support continuity of care [9].

Our results indicate a more frequent use of the Internet during clinical practice by younger pediatricians, males, and those living in Northern and Central Italy, These results are in line with the factors affecting the use of the Internet in the general population according to a National survey [18]. Our results also show a less frequent use of the Internet by family pediatricians, in agreement with the assumption that they deal with less complex clinical problems compared with specialists.

We found that younger pediatricians are more likely to be influenced in their clinical decisions by information found on the web. A possible explanation for this finding could be that older and therefore more experienced pediatricians might feel less need of a decision support system compared to younger pediatricians.

Hospital pediatricians and other pediatric profiles such as those working in private practices or local health units were also more likely to be influenced by information found online compared with family pediatricians. This observation may be linked to the greater clinical complexity of children usually seen in hospitals or in private practice as compared with primary care.

A low proportion of respondents stated that social networks may be useful for their clinical practice. We did not investigate the reasons underlying these results, however we may hypothesize that respondents were possibly concerned about privacy and confidentiality issues, as addressed in the policy statement on professionalism in the use of social media by the American Medical Association [12].

Interestingly, male respondents were more likely to use the Internet during clinical practice and to consider social networks potentially useful in clinical practice.

We could hypothesize that this is in line with a more frequent use of the Internet by males, as reported by the National Institute for Statistics [18]. Though, this result is not found in the other models. Nevertheless, this issue will deserve attention in future studies, since it might be associated with personal use of social networks in private life.

On the other hand, nearly half of respondents considered personal health records potentially useful for their practice. Registrars and hospital pediatricians, irrespective of their age, considered that integrating clinical records with information provided by the family may be useful for clinical practice. While registrars may be more oriented to new opportunities for data collection from patients and their families, hospital pediatricians, who mostly have occasional contacts with patients, may consider personal health records as a useful tool for a comprehensive view of the patients' history.

Our survey suggests that Italian pediatricians favour the use of social networks and of other tools for communicating with families. This finding seems in contrast with other studies that show that physicians are quite sceptical about the potential benefits provided by patient-physician communication [19,25]. The opinion on the usefulness of web-based tools for communication with families may depend on the perception of a potential for an easier disease management, especially regarding chronic diseases, as suggested by another study [26]. Interestingly, the use of these tools during clinical practice, although endorsed by the American Academy of Pediatrics, poses implementation challenges regarding confidentiality, integrity, and availability of patients' data [27].

### Limitations of the study

Our study has several limitations. There might have been a strong selection bias: although an invitation to join the survey has been published on the paper issue of the Society's news magazine, which is received by all the Society's members, the email invitation, sent only to those members that had provided an email address, may have selected those who are more accustomed to the use of the Internet and who likely check their mail on a regular basis. This may have overestimated results in all the items related to the use of the Internet. Nonetheless, age and sex distribution of respondents only slightly differed from that of the general pediatric community.

Participation was on a voluntary base, and therefore participants may not represent the general population of Italian pediatricians and may be selected among those with a stronger interest toward the web. This may have reflected on the proportions of pediatricians using the Internet during clinical practice and favouring social network and personal health records, that may have been overestimated.

Moreover, this is a cross-sectional study, and no previous figures are available in order to measure a time trend.

On the other hand our survey had a high quality of responses due to the limited time requested for filling in questionnaire, with a resulting low missing value rate. Moreover, despite limitations, the study may serve as a term of comparison for future cross sectional studies on the same subject and for comparison with other health settings.

## Conclusions

Our study shows that Italian pediatricians frequently use the Internet during clinical practice, mainly for guideline and bibliographic searches. Despite an encouraging attitude towards the use of personal health records, in particular by registrars and hospital pediatricians, a general resistance to the introduction of social networks in clinical practice was found. Smartphones may represent an interesting frontier that can improve information access through the Internet in a community where this support is widely available.

## Competing interests

The authors declare that they have no competing interests.

## Authors' contributions

MR, FG and AET conceived and designed the study and drafted the manuscript. EP performed the data analysis. AGU coordinated the study and reviewed the manuscript. All authors read and approved the final manuscript.

## Pre-publication history

The pre-publication history for this paper can be accessed here:

http://www.biomedcentral.com/1472-6947/12/23/prepub
